# Minipuberty and Sexual Dimorphism in the Infant Human Thymus

**DOI:** 10.1038/s41598-018-31583-3

**Published:** 2018-09-03

**Authors:** Carlos Alberto Moreira-Filho, Silvia Yumi Bando, Fernanda Bernardi Bertonha, Leandro Rodrigues Ferreira, Christiana de Freitas Vinhas, Lucila Habib Bourguignon Oliveira, Maria Claudia Nogueira Zerbini, Glaucio Furlanetto, Paulo Chaccur, Magda Carneiro-Sampaio

**Affiliations:** 1 Departament of Pediatrics, Faculdade de Medicina da Universidade de São Paulo, São Paulo, SP Brazil; 2 Department of Pathology, Faculdade de Medicina da Universidade de São Paulo, São Paulo, SP Brazil; 3 Instituto Dante Pazzanese de Cardiologia, São Paulo, SP Brazil

## Abstract

*AIRE* expression in thymus is downregulated by estrogen after puberty, what probably renders women more susceptible to autoimmune disorders. Here we investigated the effects of minipuberty on male and female infant human thymic tissue in order to verify if this initial transient increase in sex hormones - along the first six months of life - could affect thymic transcriptional network regulation and *AIRE* expression. Gene co-expression network analysis for differentially expressed genes and miRNA-target analysis revealed sex differences in thymic tissue during minipuberty, but such differences were not detected in the thymic tissue of infants aged 7–18 months, i.e. the non-puberty group. *AIRE* expression was essentially the same in both sexes in minipuberty and in non-puberty groups, as assessed by genomic and immunohistochemical assays. However, *AIRE*-interactors networks showed several differences in all groups regarding gene-gene expression correlation. Therefore, minipuberty and genomic mechanisms interact in shaping thymic sexual dimorphism along the first six months of life.

## Introduction

It is widely known that in human populations females are more susceptible to autoimmune diseases than males. Dragin *et al*.^1^ have shown that estrogen-mediated downregulation of *AIRE* (autoimmune regulator) influences sexual dimorphism in autoimmune diseases. However, this study did not cover infants along the first 6 months of age, i.e. during minipuberty^2^, a period when sex hormones conceivably act on thymic tissue. Almost concomitantly, Zhu *et al*.^3^ used quantitative RT-PCR for measuring *AIRE* expression in thymus explants surgically obtained from age-matched male-female pairs infants <6 months of age and claimed to find a consistently higher expression of *AIRE* in male infants. On the other hand, Dumont-Lagacé *et al*.^4^ showed in a murine model that sex hormones have pervasive effects on thymic epithelial cells (TEC) and that androgens have a greater impact on TEC transcriptome than estrogens. In this study, the authors observed that sex steroids repressed the expression of tissue-restricted antigens but did not alter the expression of *Aire*.

In order to further investigate the presumptive sexual dimorphism induced by minipuberty on infant thymus, we performed comparative genomic, immunohistochemical and histomorphometric studies on thymic surgical explants (corticomedullar sections) obtained at cardiac surgery from karyotypically normal male (M) and female (F) infants during minipuberty, here termed MM and MF groups. The same studies were conducted on thymic explants obtained from karyotypically normal M and F non-puberty (N) infants aged 7 to 18 months, the NM and NF groups. Analyses included gene co-expression networks (GCN) for differentially expressed genes, miRNA-target analyses, *AIRE*-centered gene-gene interaction networks encompassing the genes coding for AIRE interactors, quantitative RT-qPCR and immunohistochemical measurements of AIRE expression, and comparative thymic histomorphometry.

We employed a network-based approach for GCN analysis that allows the identification of modular transcriptional repertoires (communities) and the interactions between all the system’s constituents through community detection^5,6^. MiRNA-target analysis was used to investigate how the abundantly expressed thymic miRNAs modulate the expression of highly connected genes (hubs) in the GCNs. *AIRE*-centered networks allowed the measuring of gender-related differences in AIRE-interactors gene-gene expression correlation. Comparative histomorphometric analysis for age group and gender included measurements of thymic cortical, medullary, and lobule areas. We report changes in GCN’s hub hierarchy and in miRNA-hub interactions between MM and MF groups, whereas the expression of *AIRE* mRNA and AIRE protein showed no significant differences between MM and MF groups. However, *AIRE*-interactors networks disclosed relevant differences in all groups (M and N) regarding gene-gene expression correlation values. Differentially expressed genes (M *vs* F) were found in minipuberty groups only.

## Results

### Global gene expression and miRNA expression in minipuberty and non-puberty groups

DNA microarray technology was used to obtain mRNA and miRNA expression profiles in minipuberty (M) and non-puberty (N) groups. We identified 12,671 and 11,869 valid GO - Gene Ontology - annotated genes for M and N groups, respectively. Male (MM) and female (MF) minipuberty groups, and male (NM) and female (NF) non-puberty groups, were compared for differences among global gene expression values. The comparisons MM *vs* MF and MF *vs* NF showed significant differences (p < 0.01): global gene expression in MF was lower than in MM and NF. (see Supplementary Fig. S1a). For the miRNA expression analysis, only miRNA expression values ≥ 1.0 were considered. The minipuberty groups (MM and MF) expressed 1,198 valid miRNAs and the non-puberty groups (NM, NF) expressed 1,403 valid miRNAs. In the MM group, 1,188 miRNAs were hypo-expressed and 10 were hyper-expressed (Mann Whitney Wilcoxon test, p < 0.01). In the NM group, 959 miRNAs were hypo-expressed and 444 were hyper-expressed (Mann Whitney Wilcoxon test, p < 0.01). The comparison of mean miRNA expression values revealed two relevant differences: MM *vs* MF (p = 0.02) and MF *vs* NF (p < 0.01). The higher global miRNA expression in MF (see Supplementary Fig. S1b) corresponds to the lower level of gene expression found in this group.

A set of 16 miRNAs was found to be abundantly expressed in the minipuberty groups (see Methods), and all were hyper-expressed in the MF group (female infants). In the non-puberty groups 20 miRNAs were abundantly expressed – 15 of which also present as abundantly expressed in the minipuberty groups - and all were hyper-expressed in the NM group (male infants). The 15 abundantly expressed miRNAs in minipuberty and non-puberty groups are: miR-8069, miR-7975, miR-4459, miR-16-5p, miR-181a-5p, miR-6089, miR-7977, let-7a-5p, miR-4516, miR-3960, miR-15b-5p, miR-150-5p, miR-6869-5p, miR-342-3p, let-7b-5p. The miR-494-3p is abundantly expressed only in minipuberty groups. Five miRNAs are abundantly expressed only in the non-puberty groups: miR-205-5p, miR-let7f-5p, miR-125b-5p, miR-let7g-5p, and miR-100-5p.

### Differential Gene Expression analyses

TMEV software^7^ and significance analysis of microarrays (SAM) test^8^ was used for determining statistically significant gene expression differences. In the MM vs MF group comparison 494 differentially expressed (DE) genes were identified, all being hyper-expressed in the MM group (fold ≥1.90). No differentially expressed genes were found in the NM vs NF group comparison. Since no DE genes were found for the non-puberty (N) groups, we conducted a subsequent evaluation of all GO genes in NM and NF groups by weighted gene co-expression network analysis (WGCNA). This analysis revealed that all eigengene modules lacked a significant correlation with gender (see Supplementary Fig. S2).

### Gene co-expression network (GCN) analyses for minipuberty groups

Differentially expressed (DE) GO annotated gene co-expression networks were constructed for MM and MF groups based on gene-gene Pearson’s correlation method and the Networks 3D software^9^. A 0.960 link-strength cut-off was adopted for the MM-DE and a 0.950 link-strength cut-off for the MF-DE networks. The GCN correlation thresholds were chosen in order to ensure that most of nodes continued to be connected to the network major components (i.e. major transcriptional modules) and that the network remained stable (based on network connectivity and modularity measures) along a threshold range, i.e., maintaining network’s topological structure^10^. A set of simulations run with slightly different link-strength thresholds (from 0.900 up to 0.990) did not reveal alterations in topological structure. The resulting networks had 392 genes and 986 links for MM group and 326 genes and 946 links for MF group. All networks presented a scale-free node degree distribution. Network connectivity values were 5.03 for MM-DE network and 5.80 for MF-DE network, thus indicating similar network robustness^11^.

### Identification of high-hierarchical genes, community detection and coarse-grained community structure analysis

**Figure 1:**
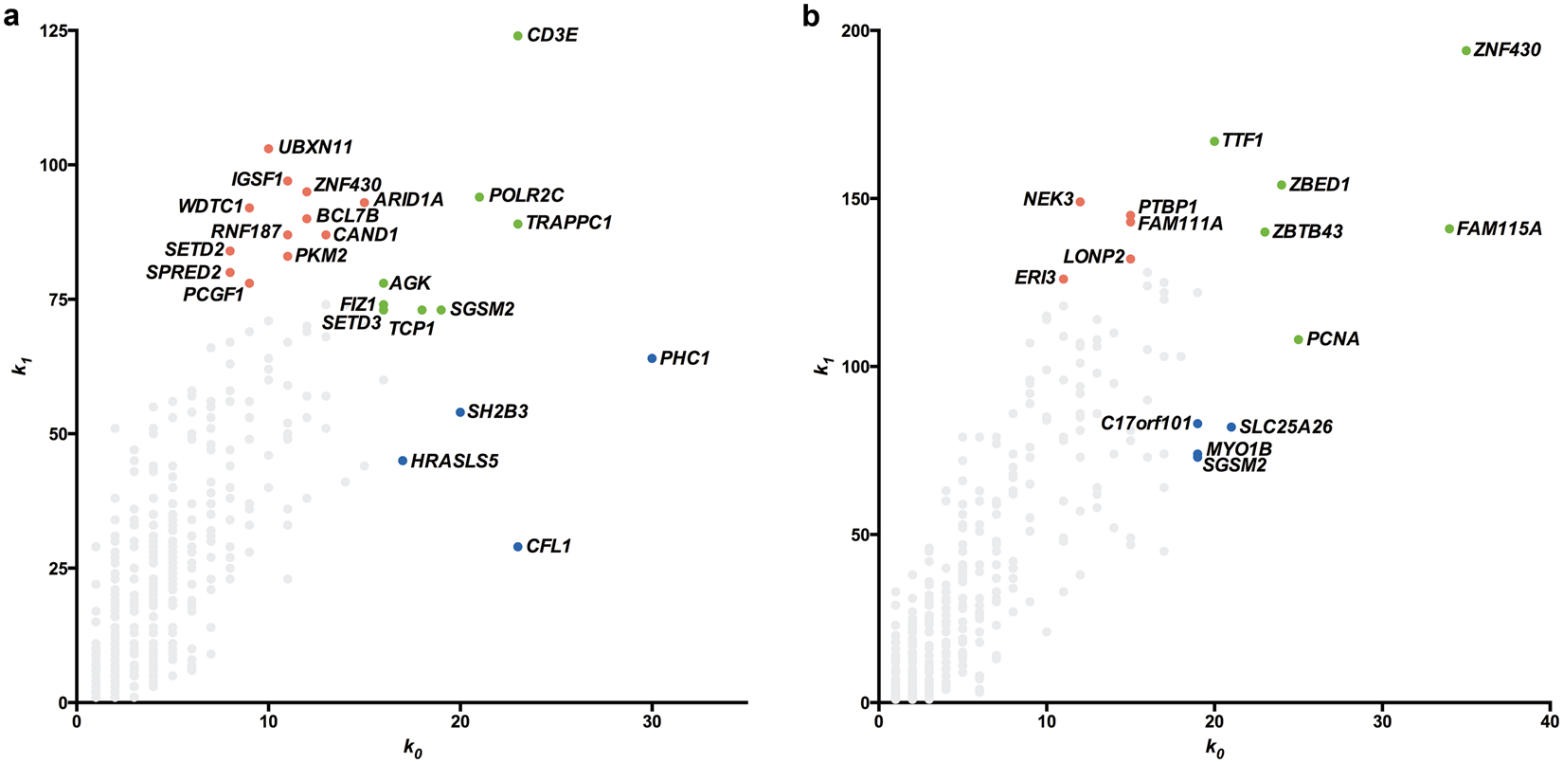
Node categorization for DE networks. Scatter plots of node degree (*k*_0_) *versus* concentric node degree (*k*_1_) measures of GO annotated genes for MM-DE and MF-DE networks (**a**,**b**). Hubs (blue), VIPs (red) and high-hubs (green) are identified by their gene symbols.

Node hierarchical categorization (hubs, VIPs, high-hubs) was accomplished using the usual node degree (*k*_0_) and the first neighborhood concentric node degree (*k*_1_), as previously described^10^. In a summarized way, there are three categories of high-hierarchy (HH) genes: hubs are highly connected nodes, VIPs (or Very Important Persons) have low number of connections but connect mostly with hubs, and high-hubs have high number of connections with highly connected nodes^10,12^. Node categorization is portrayed in Fig. 1a,b for MM-DE and MF-DE networks, respectively. These categorizations and the gene functions for all high-hierarchy (HH) genes in MM-DE and MF-DE networks appear in Table 1 and are further commented in the following sections.

**Table 1:** Transcriptional modules (communities), HH genes, and miRNA interactions in the MM- and MF-DE networks.

Gene Symbol	Category	K0	K1	Comm	Molecular function (GO)	Biological process (GO)	Abundantly expressed miRNAs^£^
**MM-DE**							
*CD3E*	High-hub	23	124	B	SH3 domain binding; T cell receptor binding	T cell receptor signaling pathway	
*POLR2C*	High-hub	21	94	B	DNA binding; RNA polymerase II activity	DNA repair	let-7b-5p
*TRAPPC1*	High-hub	23	89	B	Rab guanyl-nucleotide exchange factor activity; ER to Golgi vesicle-mediated transport	positive regulation of GTPase activity	let-7b-5p, miR-15b-5p
*AGK*	High-hub	16	78	B	ATP binding; acylglycerol kinase activity	glycerolipid metabolic process	miR-16-5p
*FIZ1*	High-hub	16	74	D	receptor tyrosine kinase binding	positive regulation of protein phosphorylation; transcription, DNA-templated	
SGSM2*	High-hub	19	73	B	GTPase activator activity; Rab GTPase binding	late endosome to Golgi transport	
*TCP1*	High-hub	18	73	B	ATP binding; ubiquitin protein ligase binding	‘*de novo*’ posttranslational protein folding	miR-15b-5p, miR-16-5p, miR-342-3p
*SETD3*	High-hub	16	73	E	histone methyltransferase activity (H3-K4 specific)	histone H3-K36 methylation	
*PHC1*	Hub	30	64	A	DNA binding; chromatin binding	histone ubiquitination	
*CFL1*	Hub	23	29	G	actin binding; protein binding	actin cytoskeleton organization	
*SH2B3*	Hub	20	54	A	phosphate ion binding; signal transducer activity	intracellular signal transduction	
*HRASLS5*	Hub	17	45	F	protein binding; transferase activity, transferring acyl groups	metabolic process	
*UBXN11*	VIP	10	103	B	ubiquitin binding	proteasome-mediated ubiquitin-dependent protein catabolic process	
*IGSF1*	VIP	11	97	B	coreceptor activity	regulation of transcription, DNA-templated	miR-16-5p
ZNF430*	VIP	12	95	B	DNA binding	transcription, DNA-templated	miR-342-3p
*ARID1A*	VIP	15	93	B	DNA binding	chromatin remodeling	
*WDTC1*	VIP	9	92	B	histone deacetylase binding	post-translational protein modification	
*BCL7B*	VIP	12	90	B	actin binding; protein binding	Wnt signaling pathway; apoptotic process	
CAND1**	VIP	13	87	B	TBP-class protein binding	protein ubiquitination	
*RNF187*	VIP	11	87	B	ubiquitin-protein transferase activity	protein autoubiquitination	miR-181a-5p
*SETD2*	VIP	8	84	I	histone-lysine N-methyltransferase activity	chromatin organization	
*PKM2*	VIP	11	83	B	MHC class II protein complex binding		
*SPRED2*	VIP	8	80	B	protein kinase binding	regulation of protein deacetylation	
*PCGF1*	VIP	9	78	B	protein C-terminus binding	histone H2A monoubiquitination	
**MF-DE**							
ZNF430*	High-hub	35	194	D	DNA binding	transcription, DNA-templated	miR-342-3p
*FAM115A*	High-hub	34	141	C	ion channel binding	regulation of anion channel activity	
PCNA**	High-hub	25	108	A	DNA polymerase binding; chromatin binding	DNA repair	
*ZBED1*	High-hub	24	154	B	DNA binding	metabolic process	miR-150-5p
*ZBTB43*	High-hub	23	140	A	DNA binding	transcription, DNA-templated	
*TTF1*	High-hub	20	167	A	chromatin binding	chromatin remodeling	
*SLC25A26*	Hub	21	82	A	transmembrane transporter activity	S-adenosyl-L-methionine transport	miR-342-3p
*C17orf101*	Hub	19	83	B	dioxygenase activity	oxidation-reduction process	
*MYO1B*	Hub	19	74	E	ATP binding; actin filament binding	actin filament organization	
SGSM2*	Hub	19	73	E	GTPase activator activity; Rab GTPase binding	positive regulation of GTPase activity	
*NEK3*	VIP	12	149	C	protein serine/threonine kinase activity	protein phosphorylation	
*PTBP1*	VIP	15	145	D	poly(A) RNA binding	alternative mRNA splicing, via spliceosome	miR-15b-5p
*FAM111A*	VIP	15	143	B	protein binding	DNA replication	
*LONP2*	VIP	15	132	D	protease binding	protein import into peroxisome matrix	
*ERI3*	VIP	11	126	B	poly(A) RNA binding	DNA catabolic process, exonucleolytic	

^*^HH genes in both networks; ^**^AIRE interactors; Comm: Community; GO: Gene Ontology; ^£^Validated interactions.

**Figure 2:**
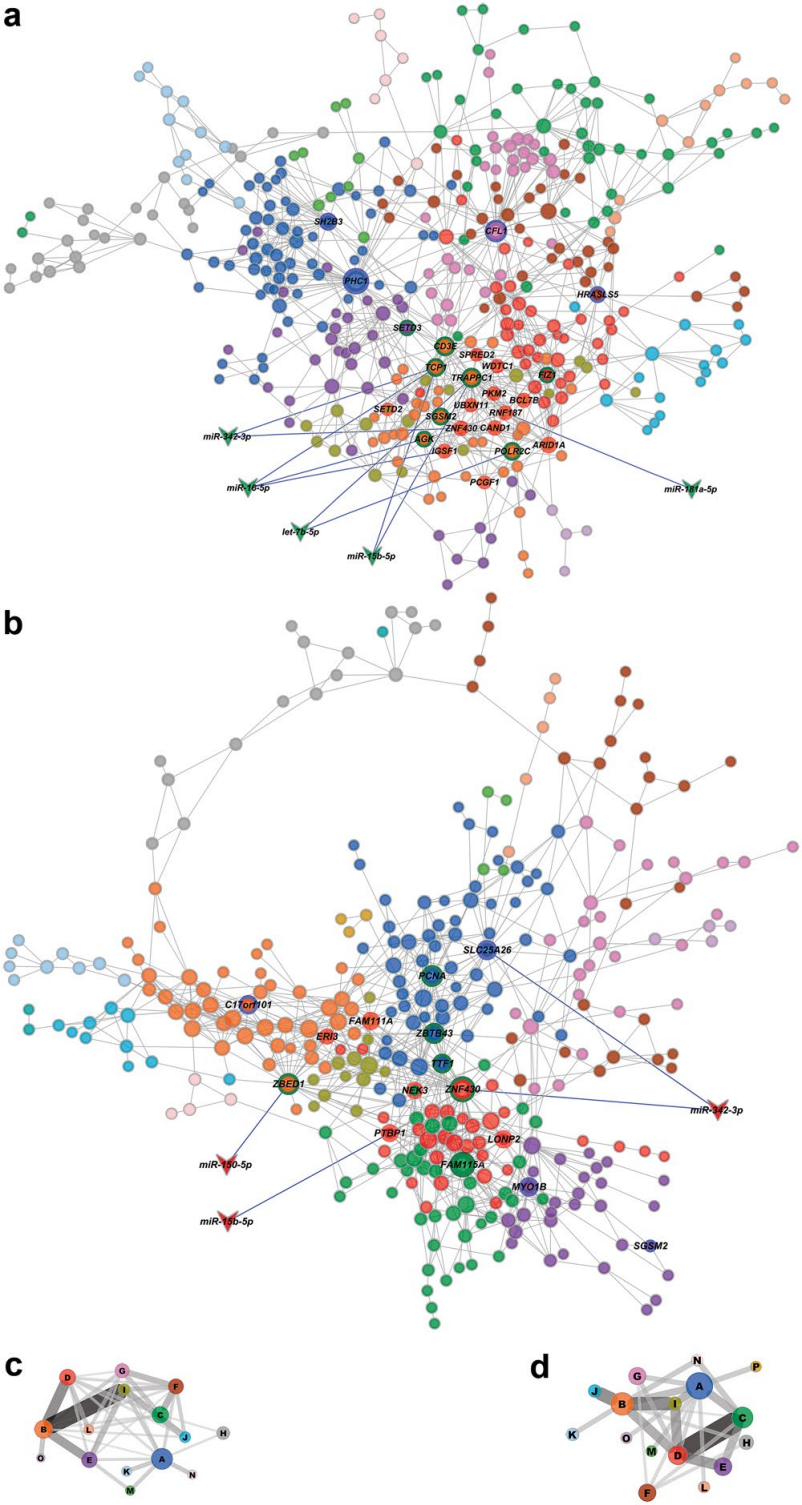
DE networks with their respective gene communities (modules), miRNA-target interactions and coarse-grained community structure (CGCS) diagrams. Network topology and community structure for minipuberty DE networks (**a** for MM and **b** for MF), and CGCSs for minipuberty DE networks (**c** for MM and **d** for MF) considering 15 and 16 communities per network, respectively. High hierarchy genes are identified by their node border color: green for high-hubs, red for VIPs, and blue for hubs. Abundantly expressed miRNAs are depicted as vee nodes. Gray lines indicate gene-gene links, whereas miRNA-gene validated interactions are indicated by blue lines. The vees filled with red or green colors indicate, respectively, hyper- or hypoexpressed miRNAs. Gene communities in both networks diagrams are distinguished by different node colors. In CGCS the communities are identified by different colors and the edge width and intensity is proportional to the connection weight of edges linking distinct communities. In the networks the node size is proportional to the number of gene-gene links. In CGCS diagrams the node size is proportional to the number of nodes/genes in each community. In the MM-DE network the communities harboring high hierarchy genes are identified by the following colors: A, blue; B, orange; D, red; F, brown; G, pink and I, olive green. In MF-DE communities’ colors are: A, blue: B, orange; C, green; D, red, and E, purple.

Figure 2a,b show the two minipuberty networks, MM-DE and MF-DE, their gene communities (modules) and the HH genes for each network. Different node colors identify the distinct gene communities in each network. Modularity values and the number of communities in each network were quite close: 0.728 and 15 communities in the MM-DE and 0.649 and 16 communities in MF-DE.

Coarse-grained community structure (CGCS) was obtained for each DE network, disclosing the relationships between each community in the network (Fig. 2c,d for MM-DE and MF-DE, respectively). Communities having the highest node strength (total probability for community’s nodes to connect to distinct communities) hold the most significant functional interactions in the network^5,13,14^. DE network’s CGCSs are further addressed in the context of subsequent analyses on gene hierarchy, gene communities and microRNA-target interactions.

### MiRNA target analysis and integrative network analysis (miRNA-HH genes)

As mentioned before, 16 miRNAs were abundantly expressed in the minipuberty groups (MM and MF). The integrative network analyses between abundantly expressed miRNAs and target HH genes from MM-DE and MF-DE networks appear in Fig. 2a,b and Table 1. All these microRNA-target interactions were experimentally validated (see Methods) and are depicted as blue vertices in Fig. 2. Here is worth to note that all miRNAs interacting with HH genes in the MM-DE and MF-DE networks play important roles in the regulation of immune processes, and particularly in the thymic environment. Let-7 miRNAs regulate NKT cell differentiation^15^. The cluster miR15/16 enhances the induction of regulatory T-cells by regulating the expression of Rictor and TOR^16^. MiR-150 controls the Notch pathway and influences T-cell development and physiology^17^. MiR-181 enhances cell proliferation in medullary thymic epithelial cells via regulating TGF-β signaling^18^ and is involved in the positive and negative selection of T-cells^19^. MiR-342-3p is a well-known regulator of the NF-κB pathway^20^, whose activation was shown to be necessary for the thymic expression of *Aire* in mice^21,22^.

In the following two paragraphs we present an overview of the functional role of the HH genes - hubs, VIPs and high-hubs – found in MM-DE and MF-DE networks, addressing their validated interactions with abundantly expressed miRNA and the CGCS analyses. Table 1 shows for all HH genes in each network: i) community distribution; ii) associated molecular functions and biological processes, in accordance with Gene Ontology (GO) categories; and iii) the validated interactions with abundantly expressed miRNAs.

### MM-DE network and microRNA-target interactions

In the MM-DE network (Fig. 2a; Table 1) community B harbors most of the HH genes (17 out of 24) and all the interactions between HH genes and abundantly expressed miRNAs. Moreover, all the HH genes in community B are VIPs (11 genes) or high-hubs (six genes), which means that these genes play relevant roles regarding the network functioning and robustness^23^. Indeed, VIPs connect different gene communities^10^ and high-hubs are essential for the maintenance of network robustness^24^. Network biology studies have shown that GCNs can be effectively used to associate highly connected genes (i.e. GCN hubs) with biological functions/processes in cells and tissues^25,26^. Actually, targeted hub attacks in protein-protein and gene-gene networks have been used to disclose relevant functional genes in health and disease^26–28^. Therefore, GCN hubs are relevant both for network topology and cell functioning.

Noteworthy, miRNA-target interactions involved only VIPs and high-hubs in MM-DE network. One of these high-hubs, *TCP1*, which codes for a molecular chaperone required for the transition of double negative to double positive T cells in the thymus^29^, has interactions with three abundantly expressed miRNAs, all exerting known regulatory roles in the immune system, as mentioned before. Functionally, most of the HH genes in MM-DE network are related to DNA and chromatin binding, DNA repair, histone modification, and ubiquitination. CGCS analysis shows clearly that community B holds the highest connection weights, thus evidencing its importance for network functioning (Fig. 2c).

### MF-DE network and microRNA-target interactions

In the MF-DE network (Fig. 2b; Table 1) the HH genes are quite evenly distributed among five gene communities: A (three high-hubs and one hub), B (two VIPs, one high-hub and one hub), C (one high-hub and one VIP), D (two VIPs and one high-hub), and E (two hubs). Abundantly expressed miRNAs were found to interact with two high-hubs, one VIP and one hub. The genes involved in these interactions were related to DNA binding (two genes), alternative mRNA splicing (one gene), and transmembrane (mitochondrial) transporter activity (one gene). The most represented molecular functions and biological processes among HH genes in MF-DE network are related to DNA binding, control of gene expression and DNA repair and replication. CGCS analysis shows that the five gene communities harboring HH genes are also the ones presenting the highest connection weights (Fig. 2d).

### *AIRE* expression assessment by microarray analysis, RT-qPCR and immunohistochemistry (IHC)

**Figure 3:**
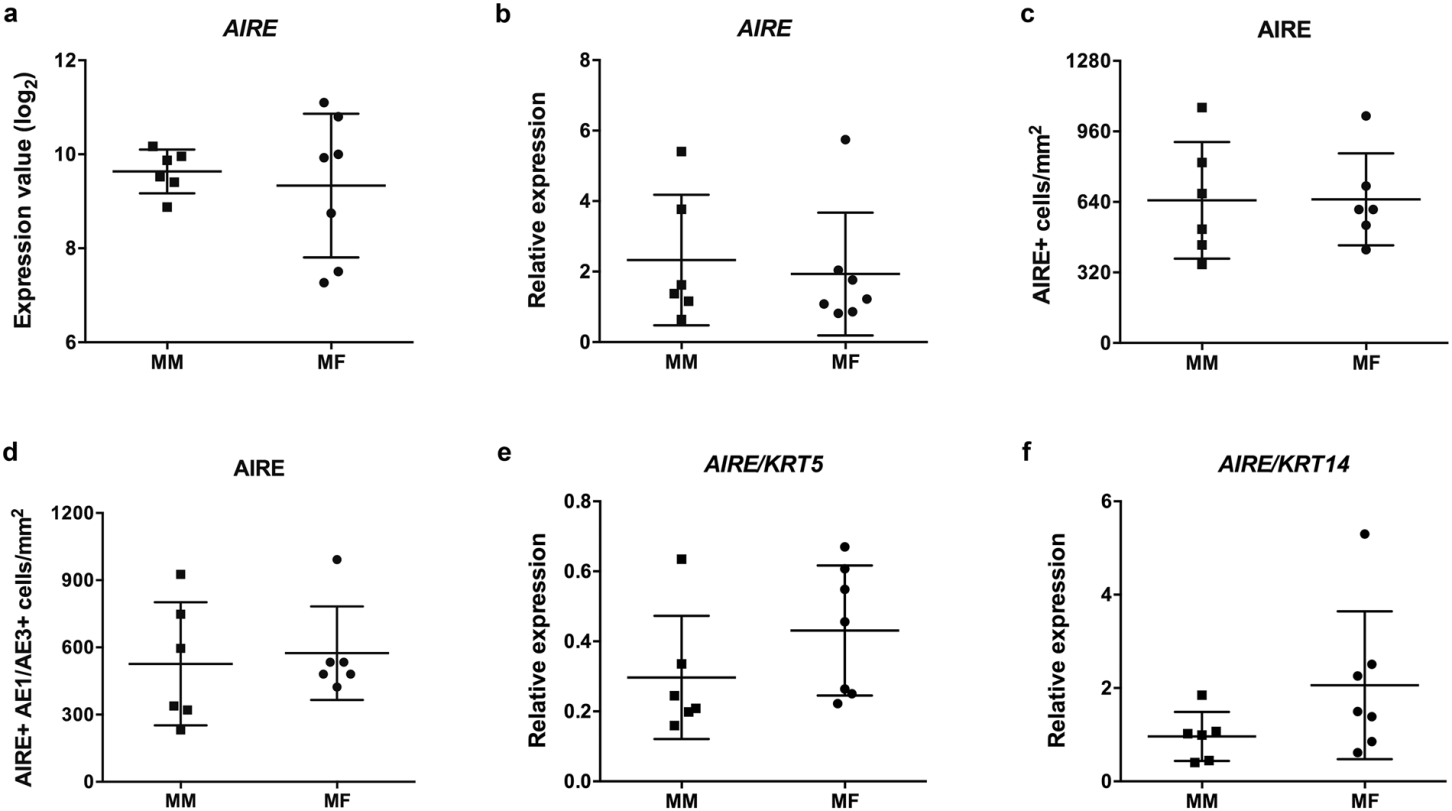
Genomic and immunohistochemical analyses of AIRE expression. DNA microarray expression values of *AIRE* (**a**) RT-qPCR relative expression of *AIRE* mRNA (**b**) Scatter plot of total AIRE positive thymic cells/mm^2^ (**c**) Scatter plot of AIRE/cytokeratin positive, mTEC cells/mm^2^ (**d**) DNA microarray relative expression of *AIRE* mRNA normalized to *KRT5* (**e**) and *KRT14*. (**f**) Unpaired Mann Whitney test was used for the comparisons shown in **a**,**e**, and **f**. Unpaired Student’s t-test was used for the comparisons shown in **b**,**c**, and **d**. Error bars represent s.d. Statistical significance was considered with p values less than 0.05.

*AIRE* expression values in MM and MF groups showed no significant difference in microarray data (p = 0.50) and in subsequent RT-qPCR analysis (p = 0.35) as shown in Fig. 3a,b, respectively. The total number of thymic AIRE-positive cells and of medullary thymic epithelial cells (mTECs) expressing AIRE – positive for AIRE and positive for the cytokeratin markers AE1/AE3 – were comparatively assessed by IHC in thymic samples from six male and six female donors aged <6 months (see Supplementary Fig. S3). The detailed procedures are described in the Material and Methods section. Statistical analysis showed no significant difference between male and female samples for total AIRE expression (p = 0.49) and for AIRE expression in mTECs (p = 0.37) as depicted in Fig. 3c,d. Additionally, microarray absolute values for AIRE mRNA expression were normalized to those of two thymic mTEC markers, keratin 5 (KRT5) and keratin 14 (KRT14), and no significant differences between male and female groups (p = 0.14) were found in both comparisons (Fig. 3e,f, respectively).

### *AIRE* interactors’ gene-gene expression relationships

The networks representing the gene-gene expression relationships between *AIRE* and its interactors (see below) were constructed for minipuberty (MM and MF) and non-puberty groups (NM and NF) according to Pearson’s correlation coefficient. In the human thymus *AIRE* is almost exclusively expressed in thymic epithelial cells (TECs): only a small fraction of thymic B cells, around 5%, express *AIRE* and B cells constitute just 1% of thymic lymphocytes^30^. Therefore, regarding *AIRE* expression there is no artifact in our data caused by thymocyte background. On the other hand, only genes known to be expressed in mice and/or human thymic epithelial cells (TECs) - and whose coded proteins were shown to physically associate with AIRE in TECs - were included in our *AIRE-*interactors network analysis. Hence, we restricted our analysis of *AIRE*-interactors to the set of genes coding for the Aire-targeted proteins previously identified in TECs by Abramson *et al*.^31^ (see Methods).

**Figure 4:**
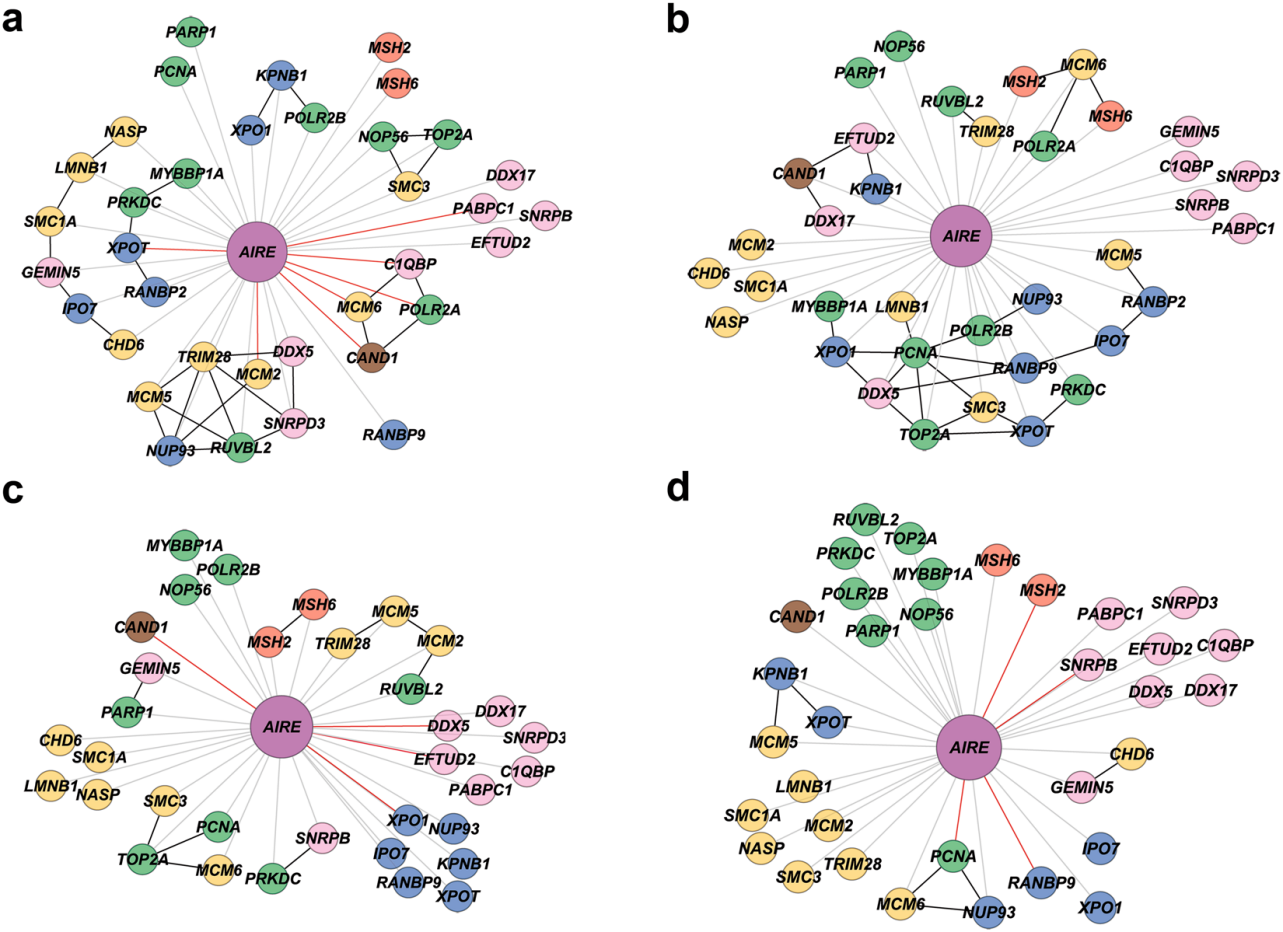
AIRE interactors’ gene-gene expression relationships. Gene-gene expression relationship networks for MM (**a**), MF (**b**), NM (**c**), and NF (**d**) groups. Nodes are colored according to their molecular function (GO): green for transcription, yellow for chromatin binding/structure, blue for nuclear transport, brown for ubiquitination, pink for pre-mRNA processing, red for DNA repair, and purple for *AIRE*. *AIR*E-gene expression correlation values < |0.70| are depicted with grey links; *AIRE*-gene expression correlation values ≥ |0.70| are depicted with red links; gene-gene expression correlation values ≥ |0.90| are depicted with black links.

**Figure 5:**
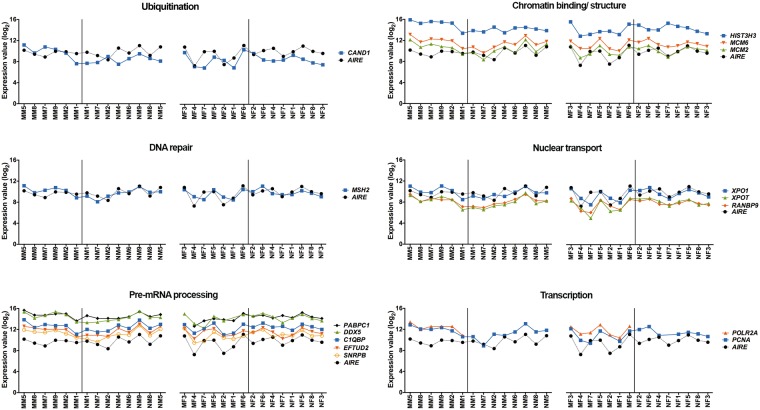
Gene expression profiles of *AIRE* interactors. Gene expression profiles of *AIRE* interactors with a Pearson’s correlation coefficient value ≥ 0.70 at least in one group, across minipuberty and non-puberty samples.

These AIRE-interactors networks included *AIRE* and other 34 genes (34 genes in the minipuberty group or 33 genes in the non-puberty group, see Supplementary Table S1), which code for proteins that are associated, directly or indirectly, with AIRE (Fig. 4) and exert impact on its functions (see Methods). *AIRE* interactors were classified according to their molecular function and represented by different node colors in the networks. Average gene expression values of all *AIRE* interactors for each group and the results of statistical tests are shown in Table S1. Gene-gene expression relationships of *AIRE* interactors presenting a Pearson’s correlation coefficient value ≥ |0.70| at least in one group across minipuberty and non-puberty samples – here termed high interactors - are highlighted in Fig. 5 and Table 2. There are 14 high-interactors distributed among minipuberty and non-puberty groups, and, consequently, distinctive profiles of *AIRE* interactors’ gene-gene relationships for each group. The MM group encompasses more high interactors - seven out of 14 - than the other three groups. MF has just three high interactors, which also are high interactors in MM. NM harbors seven high interactors, two of them also present in MM. NF has eight high interactors, all them distinctive of this group. The subnetworks formed by highly correlated genes (r ≥ |0.90|; Fig. 4) also differ for each group. Altogether, these data suggest that sex hormones and genomic background exert their influence on *AIRE* interactors’ gene-gene expression relationship during and after minipuberty.

**Table 2:** *AIRE* interactors’ gene-gene relationships.

Gene	Function	Node color	MM	MF	NM	NF
MCM2*	Chromatin binding/structure	yellow	**0.72**	0.41	0.14	0.04
MCM6*			**0.87**	0.04	0.04	0.41
*SMC3*			0.61	0.13	0.34	0.15
*CHD6*			0.53	0.37	0.24	0.44
*LMNB1*			0.24	0.08	0.27	0.22
*MCM5*			0.23	0.39	0.21	0.04
*NASP*			0.16	0.19	0.16	0.30
*SMC1A*			0.24	0.66	0.11	0.18
*TRIM28*			0.54	0.09	0.21	0.37
MSH2*	DNA repair	red	0.49	0.27	0.21	**0.72**
*MSH6*			0.22	0.11	0.06	0.31
XPOT*	Nuclear transport	blue	**0.81**	0.03	0.19	0.24
*RANBP9*			0.67	0.18	0.52	**0.78**
*IPO7*			0.30	0.20	0.32	0.58
*KPNB1*			0.42	0.43	0.50	0.03
*NUP93*			0.63	0.10	0.20	0.46
*RANBP2*			0.63	0.03	NE	NE
XPO1*			0.25	0.23	**0.71**	0.25
C1QBP*	Pre-mRNA processing	pink	**0.83**	0.55	0.43	0.34
PABPC1*			**0.80**	0.17	0.12	0.21
*DDX17*			0.46	0.38	0.56	0.32
DDX5*			0.49	0.44	**0.79**	0.11
EFTUD2*			0.46	0.28	**0.74**	0.35
*GEMIN5*			0.10	0.39	0.22	0.13
SNRPB*			0.29	0.36	0.16	**0.72**
*SNRPD3*			0.61	0.41	0.59	0.54
*POLR2A*	Transcription	green	**0.91**	0.03	NE	NE
*MYBBP1A*			0.44	0.30	0.29	0.00
*RUVBL2*			0.44	0.02	0.25	0.64
*NOP56*			0.56	0.15	0.38	0.66
*PARP1*			0.65	0.55	0.01	0.39
PCNA*			0.23	0.32	0.38	**0.85**
*POLR2B*			0.20	0.42	0.67	0.16
*PRKDC (DNA-PK)*			0.69	0.05	0.17	0.07
*TOP2A*			0.62	0.25	0.29	0.65
CAND1*	Ubiquitination	brown	**0.96**	0.10	**0.89**	0.29

Pearson’s correlation coefficients for MM, MF, NM, and NF groups. Genes are classified according to their molecular function. ^*^Genes presenting Pearson correlation coefficient ≥0.70 at least in one group (values in bold); NE: non-expressed.

### Histomorphometric analysis

Comparative analysis for MM, MF, NM and NF groups encompassed the following measurements: average cortical thickness (µM); average diameter of the medullary region (µM); total area of the lobule (1 × 10^6^ µM^2^); area of the medullary region (×10^6^ µM^2^); medullary area/lobule area (%). Statistical analysis was made for gender (M, F) and age differences (<7 mo/≥7 mo). For all datasets, no significant differences were found (see Supplementary Fig. S4).

## Discussion

The effects of sex steroids on thymic tissue constitute a matter of great interest since these hormones could act on the mechanisms of immune tolerance^1,32^. Here we investigated the effects of the transient post-natal sex steroids surge of infancy, or minipuberty, on human thymus and on AIRE expression. Comparative genomic, immunohistochemical and histomorphometric studies were conducted on thymic explants obtained from the minipuberty groups (M), i.e. from male (MM) and female (MF) children below 6 months of age, and from the non-puberty groups (N), i.e. from male (NM) and female (NF) children aged between 7 and 18 months. Significant differences were firstly observed regarding global gene expression and miRNA expression levels (see Supplementary Fig. S1a,b): comparatively, the MF group showed a diminished gene expression level and a correspondent increase in global miRNA expression, thus indicating that the estradiol surge in minipuberty down-regulates global gene expression and that miRNAs possibly play a role in such process.

MiRNA expression analysis revealed 21 abundantly expressed miRNAs, of which 15 were present in minipuberty and non-puberty groups, thus indicating commonalities between the two age groups regarding the miRNA control of gene function robustness in the human thymus. Interestingly, all these miRNAs were hyper-expressed in the MF group and hypo-expressed in the NM group.

Differential gene expression between male and female groups was found in minipuberty only and vanished in non-puberty. WGCNA for NM and NF groups revealed that all gene modules lacked a significant correlation with gender (see Supplementary Fig. S2). The significant sex-related thymic differential gene expression in minipuberty is probably triggered by the transient hormonal surge. However, its effects on gene functioning may extend beyond minipuberty, as shown by the differences found in AIRE-interactors networks.

Differentially expressed (DE) GO annotated gene co-expression networks (GCNs) constructed for MM and MF groups clearly showed pronounced changes in high-hierarchy (HH) genes between the two groups (Fig. 1a,b). The identification of the distinct gene communities in MM and MF networks and the relationships between each community in these networks was accomplished and integrated with microRNA target analysis considering only the abundantly expressed miRNAs. The resultant networks (Table 1 and Fig. 2a–d) clearly show that abundantly expressed miRNAs interact almost exclusively with high-hubs and VIPs, i.e. with genes that are essential for network robustness (high-hubs) and for connecting gene communities (VIPs). Altogether, these results indicate that testosterone and estradiol surges in minipuberty are related to significant changes in HH genes in MM and MF networks, respectively, and that these changes are under tight control by abundantly expressed miRNAs interacting with high-hubs and VIPs. In fact, relevant thymic functions, such as the induction of regulatory T cells, are regulated by abundantly expressed miRNAs^16^. Noteworthy, all miRNAs interacting with HH genes in both networks play important roles in the regulation of immune processes, and particularly in the thymic environment, as commented in the Results section.

*AIRE* expression was comparatively assessed in minipuberty male (MM) and female (MF) groups by DNA microarray (Fig. 3a), qPCR (Fig. 3b), and immunohistochemistry (Fig. 3c,d) and no significant differences were found. These results corroborate the previous findings of Dumont-Lagacé *et al*.^4^ in mice: in spite of gene expression differences in male and female thymic epithelial cells, the expression of *Aire* was found to be quantitatively the same in male and female mice thymuses. On the other hand, our data do not support the claim by Zhu *et al*.^3^ of a consistently higher expression of *AIRE* in male infants along minipuberty. Gender differences in AIRE expression were well established for prepubescent, pubescent, and adult individuals by Dragin *et al*.^1^.

A relevant finding of the present work is derived from the analysis of gene-gene expression relationships between *AIRE* and its interactors depicted in networks constructed for minipuberty (MM and MF) and non-puberty groups (NM and NF) according to Pearson’s correlation coefficient (Fig. 4). Considering the high-interactors, i.e. those presenting a Pearson’s correlation coefficient value ≥ 0.70 at least in one group across minipuberty and non-puberty samples (Fig. 5 and Table 2), it is possible to obtain distinctive profiles of *AIRE* interactors’ gene-gene relationships for each minipuberty and non-puberty groups. These results clearly evidence that sex hormones and XY and XX genomic backgrounds exert their influence on *AIRE* interactors’ gene-gene expression relationship during and after minipuberty. Interestingly, neither the sex steroids surge during minipuberty, nor the XY or XX background, seem to promote any significant gender–related histomorphometric changes in the infant thymus, corroborating previous data^33,34^.

The fact that the gender-specific *AIRE*-interactors gene-gene relationships profiles were found for all minipuberty and non-puberty groups must be further considered here. Although sex hormones are thought to be major mediators of sexual dimorphism in the immune system, sex differences in immune response arise from a complex interplay of genomic, hormonal and environmental mechanisms, whose molecular bases remain to be fully determined^35–37^. Indeed, sex differences in the susceptibility to infectious diseases^38,39^ and in response to vaccines^40^ are quite evident in infancy (below one year of age) and in early childhood (1–4 years of age), i.e. well before puberty, thus evidencing that genetic and epigenetic factors may have a role in shaping immune system sexual dimorphism.

In conclusion, our results indicate that genomic mechanisms and postnatal hormonal influences probably act synergistically in shaping thymic sexual dimorphism along the first six months of life, but this process does not involve changes in *AIRE* expression, although may involve differences – perhaps long-lasting differences - in the interactions of *AIRE* with its partners.

## Material and Methods

### Patients and thymic tissue specimens

Thymic tissue samples were obtained from 34 karyotypically normal patients that underwent cardiac surgery at Instituto Dante Pazzanese de Cardiologia, São Paulo, Brazil. Samples from patients aged up to six months were classified as minipuberty (10 males and 7 females) and samples from patients aged 7–17 months as non-puberty (9 males and 8 females; see Supplementary Table S2). The research ethics committee of Instituto Dante Pazzanese de Cardiologia has approved this research under number 4287. All methods were performed in accordance with the relevant guidelines and regulations. Informed consents have been obtained from parents and/or legal guardians. Fresh corticomedullar sections of thymic tissue were obtained at surgery room and immediately preserved with RNA*later* (cat. no. 76106, Qiagen, Valencia, USA) for total RNA extraction, or preserved in formalin and paraffin-embedded for histological analyses.

### Total RNA extraction

Total RNA was extracted from thymus tissue explants (3–4 mm^3^) using TissueRuptor and RNeasy Lipid Tissue Kit (Qiagen). RNA quality was assessed on the Agilent BioAnalyzer 2100 (Agilent Technologies, Santa Clara, USA) and stored at −80 °C.

### Microarray hybridization

In order to determine gene expression profiles, 4 × 44 K v.2 DNA microarrays (Whole Human Genome Microarray Kit, Agilent Technologies) were used. The procedures for hybridization using the fluorescent dye Cy3 followed the manufacturer’s protocols (One-Color Microarray-Based Gene Expression Analysis - Quick Amp Labeling and miRNA Complete Labeling and Hyb Kit, Agilent Technologies). For miRNA, whole human miRNA of 8 × 60 K DNA microarrays (Human miRNA Microarray slide, G4872A, Agilent Technologies), containing probes for 2,549 human miRNAs based on miRBase database (release 21.0) were used. The images were captured by the reader Agilent Bundle according to the parameters recommended for bioarrays and extracted by Agilent Feature Extraction software version 9.5.3 for gene expression and 10.7.3 for miRNA expression. Spots with two or more flags (low intensity, saturation, controls, etc.) were considered as NA, that is, without valid expression value. The R software version 2.11.1^41^ and an in house script were used for: i) sample grouping (MM *vs* MF subgroups - or NM *vs* NF subgroups); ii) excluding transcript spots presenting three or more NAs per group; iii) converting gene expression values to log base 2.

The abundantly expressed miRNAs for minipuberty and non-puberty groups were selected after analyzing miRNA expression value distribution through a scatter dot plot, thus adopting a cut-off for considering abundant expression values of 415 and 592 for MM and MF groups, respectively, and a cut-off of 343 and 325 for NM and NF groups, respectively (for detailed description of this methodology, see Supplementary Methods).

All mRNA and miRNA microarray raw data have been deposited in GEO public database (http://www.ncbi.nlm.nih.gov/geo), a MIAME compliant database, under accession number GSE113597 (Reference Series).

### MicroRNA-target analysis

A cross-search was done with the 16 abundantly expressed miRNAs for M groups and the HH genes, from DE networks, in two miRNA databases: miRTarBase 6.1^42^ for experimentally validated microRNA-target interactions, and TargetScan 7.1^43^, for predicted microRNA-target interactions. These microRNA-target HH gene interactions were integrated to the respective MM-DE and MF-DE networks and visualized by Cytoscape v3.0.0^44^.

### Weighted Gene Co-expression Network Analysis (WGCNA) for R

WGCNA is a method that identifies and characterizes gene modules whose members share strong co-expression^45^. A single network for global gene expression of the non-puberty group was constructed by means of the WGCNA package considering all 9,928 valid GO annotated transcripts^46^.

The gene expression matrix was analyzed and, considering a threshold for divergence in Euclidian distance >0.9%, one sample was excluded (NF7). Pearson’s correlation coefficient was used for obtaining gene co-expression similarity measures and for the subsequent construction of an adjacency matrix using soft-thresholding power β and topological overlap matrix (TOM). Soft-thresholding process transforms the correlation matrix to mimic the scale free topology. TOM is used to filter weak connections during network construction. Module identification is based on TOM and in average linkage hierarchical clustering. Modules are assigned to a color and represented by it module eigengene (ME), which is calculated by the first principal component analysis (PCA) and can be considered as representative of the gene expression profiles in the module^46^. The dynamic cut-tree algorithm was used for dendrogram’s branch selection.

### Module-trait association

We obtained the Gene Significance (GS) of the correlation between the gene and gender. The module association with gender was obtained using Pearson’s correlation and Student t-test p-value. Significant correlation were considered with p < 0.05.

### GCNs for differentially expressed GO annotated genes (DE): visualization, analysis and community detection

Gene co-expression networks for differentially expressed GO annotated genes (DE networks), were constructed for MM and MF groups based on Pearson’s correlation, as we previously described^6,10^. Networks were tested for scale free status by Kolmogorov-Smirnov (K-S) statistics, *i.e*. power law distributions in empirical data^47^. As these networks may grow larger in the number of components (e.g. hundreds or thousands) or present very intricate connections between them (such as hierarchical or modular structure), it becomes mandatory the use of complex network analysis methodology to better characterize such networks^6,12,48,49^.

Network visualization was accomplished using the Networks 3D software^9^ and the categorization of network nodes was obtained by concentric node degree using Concentric Measurements software^50^. We classified network nodes as VIPs, hubs or high-hubs by projecting all node values in a *k*_0_ (node degree) vs *k*_1_ (first level concentric node degree) graphic.

### Connectivity

The network connectivity *k* for non-directed networks was calculated by *k* = 2 L/N, where L stands for the number of edges and N for the number of nodes^10^.

### Community detection

Community detection was accomplished for DE networks applying the method proposed by Blondel *et al*.^51^, which attains good modularity values and presents excellent performance, as previously described^6^.

### Coarse-grained community structure

As a complementary analysis for the community detection, each GCN was rearranged in a new network accounting only for the relationships between each community, also known as coarse-grained community structure (CGCS)^6,52^.

### *AIRE* quantification by qPCR

Reverse transcription was performed from 1 μg of total RNA using the SuperScriptTM III First-Strand Synthesis SuperMIx (Invitrogen, Carlsbad, USA). The reaction was primed with oligo DT primers and Fast SYBR^®^ Green Master Mix in a total volume of 20 μL. qPCR was performed in the StepOnePlus™ Real-Time PCR System (Applied Biosystems, Foster City, USA). The standard curve method^53^ was used to analyze *AIRE* gene expression. *GAPDH* was used as endogenous control (see Supplementary Methods online). The PCR primers for *AIRE* were as follows: sense 5′-GGATGACACTGCCAGTCACG-3′ and anti-sense 5′-TCATCAGAGCTGCATGTCCC-3′; and for *GAPDH*: sense 5′-ACCACAGTCCATGCCATCAC-3′ and anti-sense 5′-TCCACCACCCTGTTGCTGTA-3′.

### *AIRE*-gene expression relationships

Firstly, we selected a set of genes coding for the Aire-targeted proteins previously identified in TECs by Abramson *et al*.^31^ through coimmunoprecipitation and mass spectrometry, and whose transcripts were found in our microarray data set, designated here as AIRE interactors. The gene expression matrix of each group was used for gene-gene expression network construction based on Pearson’s correlation coefficient (values ranging from zero to |1.00|). *AIRE* interactors were classified according to their molecular function and represented by different node colors in the networks, whose visualization was achieved through Cytoscape v. 3.0.0^44^.

### AIRE immunohistochemical analysis

Paraffin-embedded 4 μm sections (see Supplementary Table S2) were mounted on glass slides and, subsequently, dewaxed, rehydrated, and submitted to heat induced antigen retrieval in pH = 6.0 citrate buffer. Endogenous peroxidase was blocked using 3% H_2_O_2_, unspecific binding sites with 1% BSA for 5 minutes and endogenous alkaline phosphatase with Block Doublestain (DakoCytomation, Carpinteria, USA) for 10 minutes. The slides were then, double stained, firstly using polyclonal rabbit anti-AIRE (1:500; sc-33188; Santa Cruz Biotechnology, Santa Cruz, USA) incubating overnight at 4 °C. The Universal LSAB+ Kit/AP (DakoCytomation) and new fuchsin chromogen was used for color development. Sections were then incubated with monoclonal mouse anti-cytokeratin AE1/AE3 (1:1600; DakoCytomation), for 1 hour at 37 °C. The NovoLink™ Polymer Detection System (Novocastra Laboratories Ltd, Newcastle Upon Tyne, UK) and the substrate DAB was used as chromogen. Tissues were counterstained with Harris Hematoxylin. Negative controls - obtained by omitting the primary antibody incubation step - were used in every reaction.

Fifteen random areas of the medullary region of each thymus were identified using an Olympus CX31 microscope and captured with a Canon EOS Rebel SL1 digital camera. The images were analyzed with the AxioVision 4.8 program in 40X objective. Counting of positive cells was performed using Image-Pro Plus software v.4.0 (Media Cybernetics, Silver Spring, USA). AIRE-positive cells and medullary thymic epithelial cells (mTEC) expressing nuclear AIRE (AIRE-positive and AE1/AE2-positive) were expressed as cells/mm².

### Histomorphometric analysis

Paraffin-embedded and 10 μm sections were stained with haematoxylin and eosin. The histologic examination was performed with an Olympus CX31 microscope and images were captured (40x magnification) with a Canon EOS Rebel SL1 digital camera. The images were analyzed using Image-Pro Plus software v.5 (Media Cybernetics) (see Supplementary Methods online).

### Statistical analyses

Statistical analyses were performed with GraphPad Prism 6 (GraphPad Software, Inc., La Jolla, USA). Statistical significance was tested using Student’s unpaired one-tailed t-test with Welch’s correction, considering a p-value < 0.05 as significant. Immunohistochemistry and histomorphometric analysis were assessed using the nonparametric Mann-Whitney test. DNA Microarrays analyses were performed with MeV v.4.9.0^7^ using SAM (Significance Analysis of Microarrays) or Mann-Whitney-Wilcoxon test.

## Electronic supplementary material

Supplementary information

## Data Availability

The datasets generated during and/or analyzed during the current study are available from the corresponding author on reasonable request.
