# Supplementary material for: Minipuberty and Sexual Dimorphism in the Infant Human Thymus
Source: Sci Rep. 2018 Sep 3;8:13169. doi: 10.1038/s41598-018-31583-3 (PMC6120939; doi:10.1038/s41598-018-31583-3)
Supplement: Supplementary file 1 — Supplementary information [file 41598_2018_31583_MOESM1_ESM.pdf]

Minipuberty and Sexual Dimorphism in the Infant Human Thymus

Carlos Alberto Moreira-Filho[1,*], Silvia Yumi Bando[1], Fernanda Bernardi Bertonha[1], Leandro Rodrigues Ferreira[1], Christiana de Freitas Vinhas[2], Lucila Habib Bourguignon Oliveira[1], Maria Claudia Nogueira Zerbini[2], Glaucio Furlanetto[3], Paulo Chaccur[3], Magda Carneiro-Sampaio[1]

[1]Departament of Pediatrics, Faculdade de Medicina da Universidade de São Paulo, São Paulo, SP, Brazil; [2]Department of Pathology, Faculdade de Medicina da Universidade de São Paulo, São Paulo, SP, Brazil; [3]Instituto Dante Pazzanese de Cardiologia, São Paulo, SP, Brazil

Corresponding author:

Prof. Dr. Carlos Alberto Moreira-Filho
Departament of Pediatrics, Faculdade de Medicina da Universidade de São Paulo
Av. Dr. Enéas Carvalho Aguiar, 647
05403-000, São Paulo, SP, Brazil
e-mail: carlos.moreira@hc.fm.usp.br

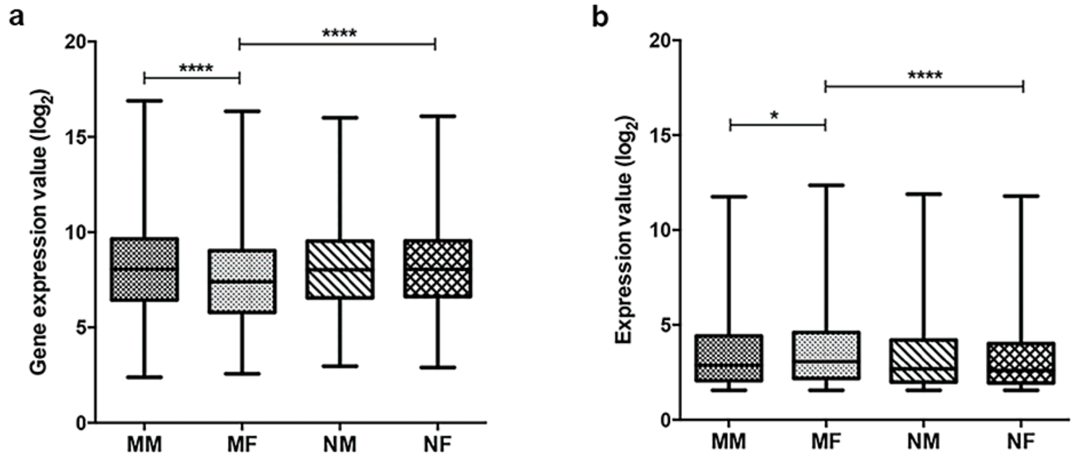

Supplementary Figure S1. Global gene expression and miRNA expression analyses. Boxplots for global gene expression (**a**) and miRNA expression (**b**), for MM, MF, NM, and NF groups. Unpaired Student’s t-test with Welch`s correction was used for the comparisons. Error bars represent s.d. *p=0.02 and ****p<0.0001.

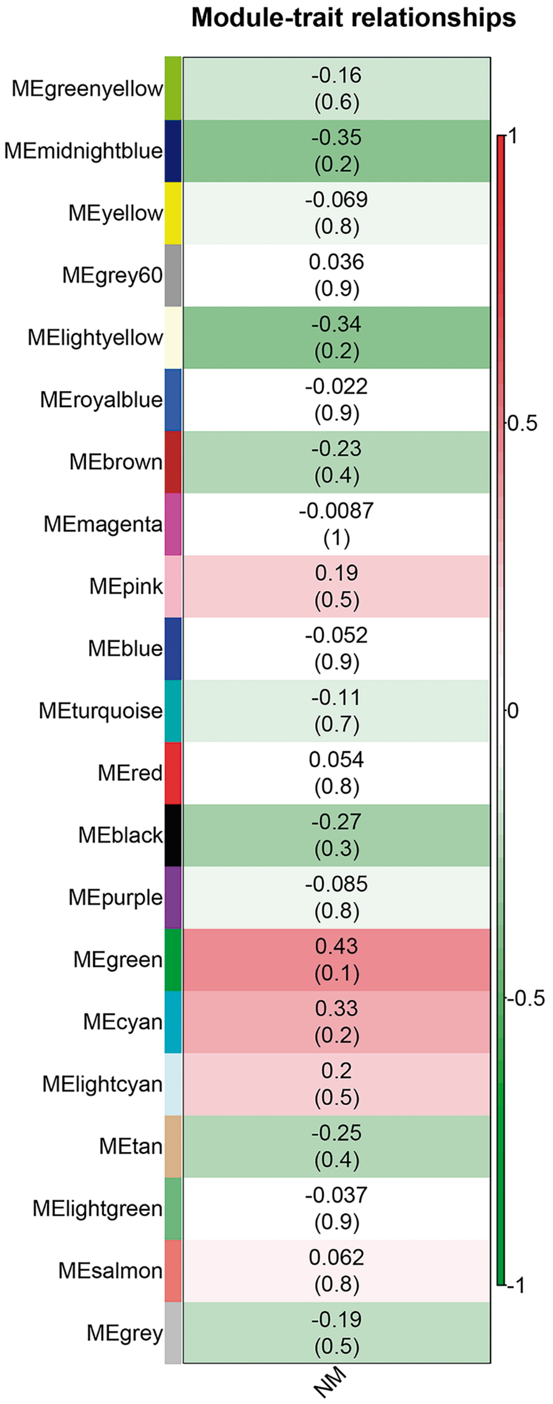

Supplementary Figure S2. WGCNA modules and Module Eigengene (ME) correlations with gender (male) for non-puberty group. In the rows, MEs are named by their module colors. In the column it is shown the trait of interest (gender). Numbers inside each colored box are the correlation coefficients between the ME and the specific trait, with p-value between brackets. The same values are true for the female group (NF), but with an opposite correlation coefficient signal. The more intense the color of the box, the more negatively (green) or positively (red) correlated is the module with the trait. None correlation presented p<0.05.

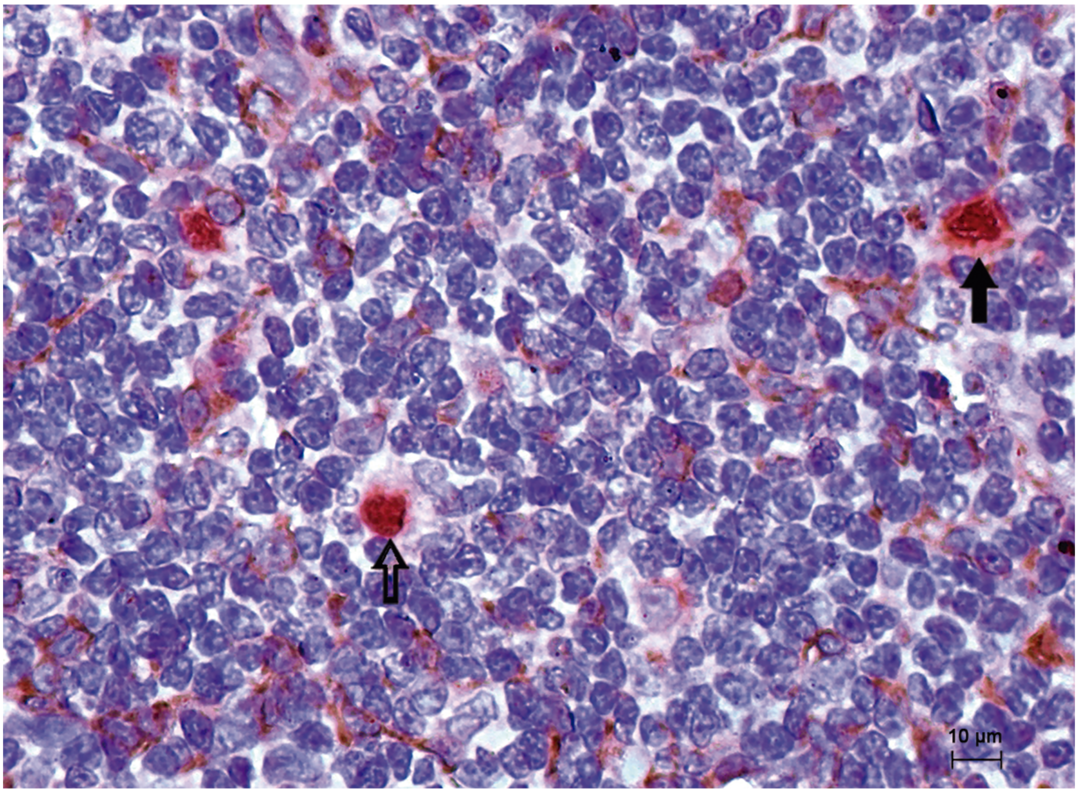

Supplementary Figure S3. Double-staining immunohistochemistry for AIRE (red color) and cytokeratin (AE1/AE3 brown color) showing AIRE/cytokeratin positive, mTEC cell (filled arrow) and AIRE-positive/cytokeratin negative, non-mTEC cells (empty arrows) cells in thymic medullary area, 400x.

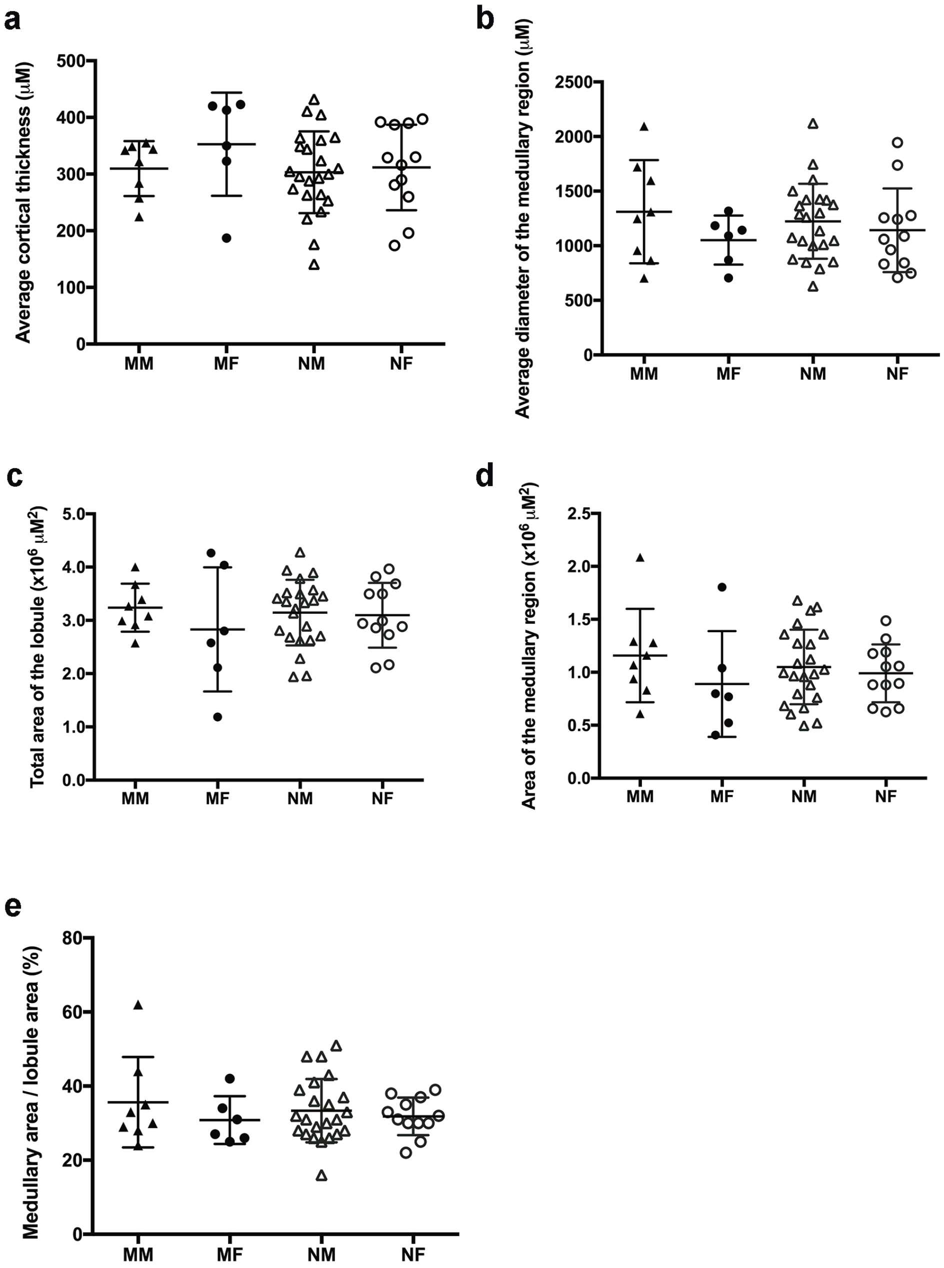

Supplementary Figure S4. Comparative histomorphometric analysis for average cortical thickness (μM) (**a**); average diameter of the medullary region (μM) (**b**); total area of the lobule ($1x10^6$ $μM^2$) (**c**); area of the medullary region ($x10^6$ $μM^2$) (**d**); medullary area/lobule area (%) (**e**). Statistical significance was considered with $p<0.05$.

Supplementary Table S1. ***AIRE* interactors` gene expression.** Gene expression average values for each group and statistically significant p-values for ANOVA or SAM.

	Gene expression average				Statistics (p-value with adjusted Bonferroni)	
**Gene**	**MM**	**MF**	**NM**	**NF**	**ANOVA**	**t-test (SAM)**
AIRE	9.64	9.31	9.80	9.96		
C1QBP	12.61	12.15	12.40	12.53		
CAND1	9.85	8.31	8.36	8.47		
CHD6	7.67	7.20	7.78	7.65		
DDX17	10.33	9.56	10.07	9.52		
DDX5	14.62	13.82	14.10	14.33		
EFTUD2	11.78	11.40	11.62	11.49		
GEMIN5	8.03	7.52	7.79	7.75		
IPO7	7.23	6.56	7.06	6.94		
KPNB1	12.59	11.88	12.24	12.11		
LMNB1	10.27	9.51	9.98	9.82		
MCM2	10.82	9.95	10.21	10.21		
MCM5	10.19	9.26	9.71	9.64		
MCM6	11.92	11.13	11.22	11.39		
MSH2	10.16	9.44	9.61	9.80		
MSH6	11.64	11.13	11.33	11.30		
MYBBP1A	7.46	6.75	7.26	7.39		
NASP	13.66	12.97	12.75	12.74		
NOP56	11.09	10.28	10.54	10.74		
NUP93	9.43	8.38	8.95	8.89	0.01	
PABPC1	14.77	13.99	14.52	14.51		
PARP1	9.66	8.89	9.32	9.46		
PCNA	11.92	10.77	11.17	11.41		
POLR2A	12.30	11.74	NE	NE		
POLR2B	10.52	9.61	10.28	10.25		
PRKDC	8.34	7.04	7.13	7.56		
RANBP2	6.91	6.23	NE	NE		
RANBP9	8.36	7.43	8.00	7.95	0.01	
RUVBL2	9.80	9.14	9.34	9.39		
SMC1A	8.55	8.43	9.05	9.35		
SMC3	11.05	10.39	10.50	10.67		
SNRPB	11.40	10.67	11.04	11.20		
SNRPD3	10.84	9.34	9.63	10.34		
TOP2A	10.50	9.41	9.71	9.87		
TRIM28	11.37	10.63	10.96	11.02		
XPO1	10.06	9.16	9.60	9.78		
XPOT	8.31	7.18	7.74	8.14		

Supplementary Table S2. Demographic data from minipuberty and non-puberty groups.

Case	Gender	Age at surgery (months)
**Minipuberty**		
**MM1**[§,*,θ]	male	6.2
**MM2**[§,*,θ]	male	6.1
**MM3**[**,θ]	male	2.4
**MM4**[**,θ]	male	2.0
**MM5**[§,*]	male	0.4
**MM6****	male	3.6
**MM7**[§]	male	1.0
**MM8**[§]	male	0.6
**MM9**[§,θ]	male	1.9
**MM10**[θ]	male	6.0
**MF1**[§,*,θ]	female	6.3
**MF2**[§,*,θ]	female	6.3
**MF3**[§,*]	female	0.2
**MF4**[§,*, θ]	female	3.6
**MF5**[§,*,θ]	female	5.3
**MF6**[§,θ]	female	6.8
**MF7**[§,θ]	female	4.4
**Non-puberty**		
**NM1**[§,*]	male	7.6
**NM2**[§,*]	male	9.1
**NM3****	male	18.3
**NM4**[§,*]	male	13.0
**NM5**[§,*]	male	17.0
**NM6**[§,*]	male	13.3
**NM7**[§]	male	8.4
**NM8**[§]	male	16.0
**NM9**[§]	male	14.0
**NF1**[§,*]	female	17.0
**NF2**[§,*]	female	14.0
**NF3**[§]**,***	female	9.9
**NF4**[§,*]	female	14.6
**NF5**[§,*]	female	8.3
**NF6**[§,*]	female	14.0
**NF7**[§]	female	15.0
**NF8**[§]	female	9.0

*MM: minipuberty male; MF: minipuberty female; NM: nonpuberty male; NF: nonpuberty female; yr: years; mo: months; d: days; [§]: samples used for gene expression analysis; *samples used for gene expression and miRNA analysis; ** samples only used for miRNA analysis; [θ]: samples used for AIRE immunohistochemistry.*

Supplementary methods

MicroRNA microarray analysis. The abundantly expressed miRNAs for minipuberty and non-puberty groups were selected after analyzing miRNA expression value distribution through a scatter dot plot, thus adopting a cut-off for considering an abundant expression of 415 and 592 for MM and MF groups, respectively, and a cut-off of 343 and 325 for NM and NF groups, respectively. The fold change values were calculated as the ratio of the average expression value of each abundantly expressed miRNA to the average expression value of the non-abundantly expressed miRNAs for each group (**Table SM1 and Figure SM1**).

Table SM1. Abundant miRNAs' average expression and fold change for minipuberty and non-puberty groups.

miRNA	Average expression				Fold change			
	MM	MF	NM	NF	MM	MF	NM	NF
miR-8069	3329	5251	3798	3550	128	159	152	154
miR-7975	2289	3536	1724	1577	88	107	69	69
miR-4459	1902	3038	479	454	73	92	19	20
miR-16-5p	1567	2459	2690	2567	60	75	108	112
miR-181a-5p	740	1180	656	641	28	36	26	28
miR-6089	709	1121	662	636	27	34	26	28
miR-7977	735	1102	765	726	28	33	31	32
let-7a-5p	694	1095	1552	1518	27	33	62	66
miR-4516	606	879	464	455	23	27	19	20
miR-3960	602	846	777	763	23	26	31	33
miR-15b-5p	589	818	1013	994	23	25	41	43
miR-494-3p	509	732	-	-	20	22	-	-
miR-150-5p	484	730	753	733	19	22	30	32
miR-6869-5p	450	623	366	343	17	19	15	15
miR-342-3p	435	621	571	553	17	19	23	24
let-7b-5p	415	592	948	929	16	18	38	40
miR-205-5p	-	-	709	680	-	-	28	30
let-7f-5p	-	-	611	568	-	-	24	25
miR-125b-5p	-	-	510	462	-	-	20	20
let-7g-5p	-	-	449	437	-	-	18	19
miR-100-5p	-	-	343	325	-	-	14	14

Average expression for abundant miRNAs was 1003, for MM; 1539, for MF; 992, for NM; 946, for NF. Average expression for non-abundant miRNAs was 26, for MM; 33 for MF; 25 for NM; 23 for NF.

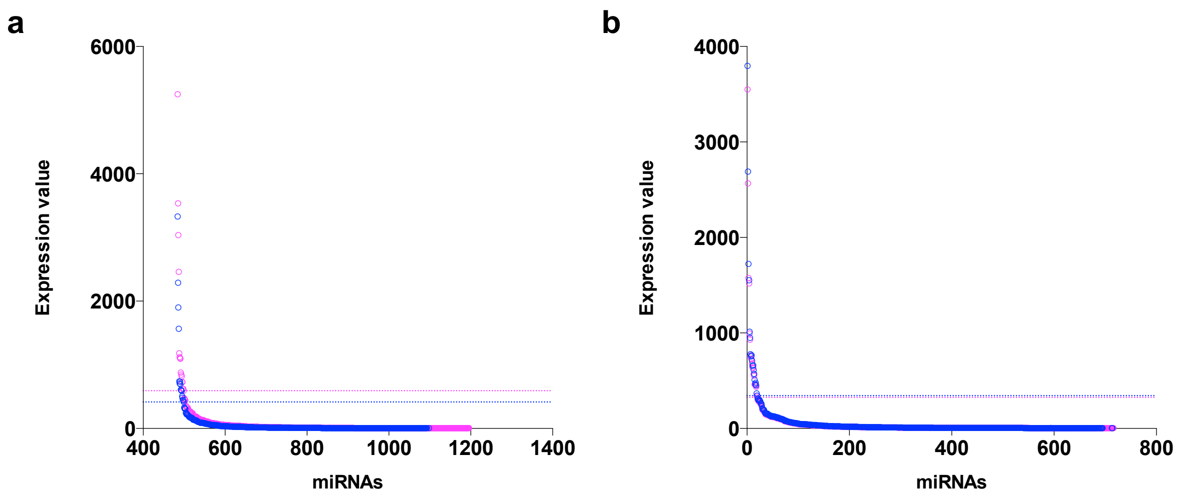

Figure SM1. Distribution of miRNAs' expression in minipuberty (**a**) and non-puberty (**b**) groups. Blue and magenta dots are referred to male and female samples, respectively. Blue and magenta dashed lines show cut-off values for abundantly expressed miRNAs' selection for male and female samples. Expression values less than 1.0 were considered background.

Histomorphometric analysis. Three images were obtained from each case and analyzed using Image-Pro Plus software v.5 (Media Cybernetics). For each photo, at 40x magnification, ten random measurements of the thickness of the cortex were obtained, and then the average of 30 measures was calculated for each case. For each case, three random areas of the lobule and the medullary region were obtained. Comparative analysis encompassed the following measurements: average cortical thickness (μM); average diameter of the medullary region (μM); total area of the lobule ($1x10^6$ μM^2); area of the medullary region ($x10^6$ μM^2); medullary area/lobule area (%). Statistical analysis was made for gender (M, F) and age differences (<7mo / ≥7mo) (**Figure S3**).
